# Editorial: Training and performance in canoe slalom

**DOI:** 10.3389/fphys.2024.1385673

**Published:** 2024-02-27

**Authors:** Leonardo Henrique Dalcheco Messias, James M. Wakeling, Filipe Antônio Barros Sousa, Ivan Gustavo Masseli dos Reis

**Affiliations:** ^1^ Research Group on Technology Applied to Exercise Physiology—GTAFE, Health Sciences Postgraduate Program, Sao Francisco University, Braganca Paulista, Brazil; ^2^ Department of Biomedical Physiology and Kinesiology, Simon Fraser University, Burnaby, BC, Canada; ^3^ Laboratory of Applied Sports Sciences, Federal University of Alagoas, Maceió, Alagoas, Brazil

**Keywords:** canoe slalom, training, kayakers, canoeing, performance

Canoe slalom presents significant scientific challenges due to its intricate nature. In this whitewater discipline, athletes navigate gates over a turbulent river course. The Olympic Games feature the K1 (kayak single) and C1 (canoe single) classes, along with the international challenge of the canoe double category (C2). In both C1 and C2 canoeing, athletes use a single-blade paddle, kneeling in the boat with their legs tucked beneath their body. Conversely, kayaking employs a double-bladed paddle with the athletes seated in the boat. Improving canoe slalom athlete performance requires interdisciplinary collaboration among biomechanists, physiologists, engineers, sports scientists, psychologists, and coaches, which was the focus of the Research Topic.

The level of attention and resources for research in any sport can vary depending on factors such as popularity, funding, and institutional support. Sprint canoeing, an Olympic discipline practiced more widely than canoe slalom in many countries, receives greater attention from researchers, sports scientists, and funding agencies. This attention results in more research studies, methodologies, and advancements in knowledge compared to canoe slalom.

For example, a search using the terms [(canoe slalom) AND (performance)] NOT (sprint) in the Web of Science database yielded 31 results. In contrast, using the terms [(sprint canoe) AND (performance)] NOT (slalom) returned over double the results, with 73 entries. This highlights the disparity in research attention between the two disciplines. Nonetheless, canoe slalom still attracts interest due to its unique challenges and potential for interdisciplinary studies focusing on athlete performance.

In an effort to address this gap, Messias et al. systematically reviewed studies that evaluated and compared mechanical, physiological, and technical parameters with athlete performance in canoe slalom. Their review included eight eligible studies involving a total sample of 117 male athletes. The studies revealed significant associations between mechanical/physiological factors and slalom performance. Most studies highlighted the importance of high-force development and a significant role of aerobic metabolism during slalom tasks. Interestingly, although aerobic metabolism is essential for performance, it may not necessarily correlate with winning medals in canoe slalom races.

The first experimental study published in this Research Topic by Wakeling et al. investigated paddle force asymmetries between on-side and off-side strokes in C1 slalom paddling. The study tested hypotheses regarding whether on-side strokes yield higher forces than off-side strokes, whether dominant and non-dominant sides produce similar forces due to assumed symmetry and whether differences in stroke forces were related to the sex of the athlete.

The authors observed that certain paddling techniques, such as switching transitions, can be influenced by the athlete’s sex. Despite the increasing adoption of switching transitions among younger male athletes, the study found that their male counterparts still preferred other techniques. Paddle forces exhibited asymmetries ranging from 14.2% to 17.1% for male and 11.1%–14.4% for female K1 kayakers. Interestingly, canoeists showed comparable asymmetry to kayakers in “on-side” strokes but significant differences in “off-side” strokes. “Off-side” stroke forces for male C1 athletes resembled their “on-side” forces, while female C1 athletes experienced significantly lower forces (ranging from −20.8% to −29.5%) on their “off-side” strokes.

This Research Topic aimed to address a notable gap in the literature concerning the limited studies involving female canoe slalom athletes. In addition to the previously mentioned study, Busta et al. contributed to this area by investigating the morphology and upper-limb strength of female canoe slalom paddlers. Their study sought to uncover potential morphological differences among various performance groups within the female cohort.

The findings indicated that female canoe slalom athletes ranked in the world’s top 10 exhibited larger arm and forearm circumferences than the rest of the elite athletes, along with a mesomorphic somatotype characterized by lower body fat and higher muscle mass. Moreover, these world’s top 10 athletes demonstrated significantly greater upper limb strength, underscoring the critical role of muscular strength in navigating the challenging canoe slalom environment. Busta et al. concluded that achieving success in female canoe slalom requires well-developed musculature, optimized strength, and minimal lower limb hypertrophy and body fat. These conclusions shed light on the physical attributes necessary for elite performance in female canoe slalom and contribute to a deeper understanding of the sport’s demands.

The last study published in this Research Topic addressed a key issue in canoe slalom research: performance testing (Manchado-Gobatto et al., 2014; Messias et al., 2015a; Messias et al., 2015b; Ferrari et al., 2017; Messias et al., 2018; Balas et al., 2020). Conducted by Vadja et al., the study assessed the test-retest reliability of four flatwater performance tests among canoe slalom athletes, both male and female. The authors developed these tests specifically for training sessions, designed to require minimal time and equipment and to be easily manageable for coaches to administer. Among international-level canoe slalom athletes, the study found that rankings had excellent relative reliability between test and retest for all four tests (ICC = 0.96–0.98). Additionally, the typical variation in test times across the four tests did not exceed 1.4%, demonstrating excellent absolute reliability.

Based on these findings, Vadja et al. recommended that coaches utilize these dependable flatwater tests to monitor changes in performance-related physical fitness over time, assess the effectiveness of training programs, and identify any asymmetries in canoe slalom athletes between their preferred and non-preferred sides. This study provides valuable insights for coaches and athletes aiming to optimize training and performance in canoe slalom.

The editors extend sincere gratitude to the esteemed authors whose invaluable contributions have enriched this discourse. This Research Topic significantly advances our understanding of canoe slalom, pushing the boundaries of knowledge and providing substantial evidence regarding critical factors in the physical conditioning of athletes in this sport. As a result, these findings hold significant implications for optimizing performance outcomes and have the potential to further expand evidence-based practices in the field.

However, there is still much to explore. Future investigations should delve into diverse fields such as training methodologies, performance assessments, and the application of mathematical models. These endeavors are essential for integrating biomechanical, physiological, nutritional, psychological, technical, and sleep-related aspects into a comprehensive understanding of athletic performance. Anticipated developments in scientific inquiry within these domains are poised to enhance our understanding and optimization of athletic performance in canoe slalom in the foreseeable future.
